# Impaired Effort Allocation in Patients with Recent-Onset Schizophrenia and Its Relevance to Negative Symptoms Assessments and Persistent Negative Symptoms

**DOI:** 10.3390/jcm11175060

**Published:** 2022-08-28

**Authors:** Ezgi Ince Guliyev, Sinan Guloksuz, Alp Ucok

**Affiliations:** 1 Department of Psychiatry, Erenkoy Training and Research Hospital for Mental and Neurological Diseases, University of Health Sciences, Istanbul 34736, Turkey; 2 Department of Psychiatry and Neuropsychology, Maastricht University Medical Centre, 6202 Maastricht, The Netherlands; 3 Department of Psychiatry, Yale School of Medicine, New Haven, CT 06510, USA; 4 Department of Psychiatry, Faculty of Medicine, Istanbul University, Istanbul 34093, Turkey

**Keywords:** effort expenditure, negative symptoms, recent-onset schizophrenia, persistent negative symptoms, motivation

## Abstract

(1) Background: Our aims in this study were (i) to compare effort allocation capacity measured between patients with recent-onset schizophrenia (SCZ) and healthy controls (HCs), (ii) within the SCZ, to investigate the association of effort allocation capacity with negative symptoms (NS), and (iii) to compare this association with the type of NS scale used. (2) Methods: Thirty-one patients with SCZ and 30 HCs participated in the study. The NS was examined using an older-generation (Scale for the Assessment of Negative Symptoms, SANS), a newer-generation (Brief Negative Symptoms Scale, BNSS), and a self-rated (Self-evaluation of Negative Symptoms Scale, SNS) negative symptom scale, as well as longitudinally by using persistent NS (PNS) distinction. (3) Results: The SCZ group was less willing to expend effort in high/moderate-probability and -magnitude conditions but more in low-probability and -magnitude conditions. A general reduction in effort allocation capacity was also present. Patients with PNS were less likely to choose hard tasks than non-PNS patients. Clinician-rated scales correlated with 50% probability and moderate-reward-magnitude conditions. Correlations with the SNS were minimal. (4) Conclusions: Our findings suggest that patients with SCZ may show a general reduction in effort allocation capacity and make inefficient choices, although they are not totally reward-insensitive. The effects of NS on effort expenditure can be more pronounced when the rewarding stimulus is vague.

## 1. Background

Negative symptoms (NS) are core features of schizophrenia (SCZ), which appear in the early stages and may persist significantly throughout the disease process [1]. They are linked to poor functional and treatment outcomes [2] and represent an unmet therapeutic need [3]. Studies showed that more than half of the individuals with SCZ have at least one NS [4,5]. Among them, motivational deficits have been consistently associated with functional or vocational impairments [6,7,8,9]. Despite their frequency and the burden they impose on patients’ lives, there are still challenges in identifying and conceptualizing motivation deficits [10,11].

Several behavioral paradigms based on reward processing mechanisms have been proposed to identify and conceptualize motivation deficits in patients with SCZ [12,13,14]. Among these, the paradigms related to effort–cost computation [15], which measure how much physical effort an individual is willing to exert to obtain varying magnitudes of reward, stand out with a more solid translational neuroscientific background [16]. Current evidence suggests that individuals with SCZ show impairments in effort allocation for rewards compared to healthy controls (HC), which means they are failing to maximize their reward by not choosing the high-effort options when the reward magnitude or probability of getting the reward is higher [17,18,19,20,21,22,23,24]. Only a few studies reported otherwise [25].

Studies examining the relationship between inefficient effort allocation and NS produced inconsistent findings. For example, there are studies reporting a negative correlation between NS measured by clinical scales and effort-based decision making paradigm performances [21,23,26,27,28], supporting the hypothesis that patients with more NS exert less effort to obtain a reward. However, some studies found only a negative trend-level correlation [19], a positive correlation [20], or no correlation [18,22,24,25,29,30,31] between NS and effort allocation capacity. NS was also investigated categorically in studies that found differences in effort expenditure performances across high- and low-NS groups [24,28,31]. Only one study considered the endurance of NS, and they found a group difference in the effort allocation between the deficit syndrome and non-deficit syndrome [29]. To our knowledge, no study has investigated effort-based decision-making differences in SCZ patients employing the proposed persistent negative symptoms (PNS) criteria [32]. 

A closer examination of mixed results reveals methodological differences between these studies. For example, task performances were sometimes correlated with NS total score [19,30], but sometimes with amotivation score [20,27]. Moreover, some studies used an older-generation scale [19,21,24,30], while others used a newer-generation scale such as the Clinical Assessment Interview for Negative Symptoms (CAINS) or the Brief Negative Symptoms Scale (BNSS) [20,22,31] or a scale specific to apathy or anhedonia [21,23,24,30]. Very few studies evaluated self-report NS [22,29]. Selection of the NS scale is particularly important as the conceptualization of NS has evolved since the development of earlier scales, and different scales might reflect/cover different aspects of the NS construct, although correlated in validation studies [1,33]. In fact, a recently published European Psychiatric Association (EPA) guidance on the assessment of NS recommended against the use of older-generation scales alone and supported the inclusion of newer-generation and self-report scales to better evaluate the experiential domains [33]. There is also no consensus on the measures of the task performance. The most consistently used ones were the rate of hard task choice in the high-reward-magnitude or high-probability trials, but other parameters were also present. The majority of the studies were conducted with chronic SCZ, which increases the likelihood of confounding factors. In fact, only one study included subjects with first-episode psychosis [24].

As it is hypothetically expected that effort motivation has a strong relationship with NS, assessments of NS and choice of the parameter that represents the effort task may have a role in these conflicting results. Therefore, in this study, we aimed to investigate effort-based decision-making in patients with recent-onset SCZ compared to HC using the Effort Expenditure for Reward Task (EEfRT) [15]. We also examined the association of NS with effort allocation capacity using an older-generation (Scale for the Assessment of Negative Symptoms, SANS), a newer-generation (BNSS), and a self-rated (Self-Evaluation of Negative Symptoms Scale, SNS) NS scale, as well as longitudinally by using PNS distinction. Lastly, we aimed to compare this association with the type of NS scale used.

## 2. Materials and Methods

### 2.1. Participants

Participants of the study were 31 patients with recent-onset SCZ recruited from Istanbul University Faculty of Medicine, Department of Psychiatry, and 30 healthy volunteers matched in terms of age, gender, and education year recruited through advertisements in the local communities. Inclusion criteria for the SCZ group were a diagnosis of SCZ according to DSM-5, clinical stabilization with antipsychotics for at least 3 months, illness duration of fewer than 5 years, age > 18, and consent to participate. Participants with a history of substance abuse in the past year, intellectual disability, a neurological disorder, or a health condition that might compromise the evaluation process or course of disease were excluded. For the HC group, in addition to the above exclusion criteria, current psychiatric diagnosis, lifelong diagnosis of psychotic disorder, and family history of psychotic disorders were also sought. Patients were also excluded if they were stabilized with a first-generation antipsychotic to minimize extrapyramidal or secondary symptoms. All patients were using second-generation antipsychotics in both interviews. Olanzapine equivalent doses were calculated according to Leucht et al. [34].

The authors assert that all procedures contributing to this work comply with the ethical standards of the relevant national and institutional committees on human experimentation and with the Helsinki Declaration of 1975, as revised in 2008. All procedures involving human subjects were approved by the Clinical Research Ethics Committee of Istanbul University Faculty of Medicine (approval number 1032). All adult participants provided written informed consent to participate in this study.

### 2.2. Clinical and Cognitive Measures

NS patients were evaluated with the BNSS [35,36], SANS [37,38], and SNS [39,40]. A categorical approach for assessing NS was also considered using the criteria proposed by Buchanan for the Persistent Negative Symptoms (PNS) [32]. Accordingly, patients with at least moderate levels of NS persisting for at least 6 months with no or mild levels of positive, depressive, and extrapyramidal symptoms were categorized into the PNS group. In this study, the persistence of NS was assessed with BNSS. To measure other symptom domains, we used the Scale for the Assessment of Positive Symptoms (SAPS) [41,42], Calgary Depression Scale for Schizophrenia (CDSS) [43,44], and Extrapyramidal Symptoms Rating Scale (ESRS) [45]. The level of psychosocial functioning was evaluated with the Personal and Social Performance Scale (PSP) [46,47]. The Brief Cognitive Assessment Tool for Schizophrenia (B-CATS) comprising Trail Making Test-B [48], Category Fluency [49], and Digit Symbol Substitution [50] tests was administered to both groups to determine their cognitive functions [51]. All clinical and cognitive assessment tools have been translated into and validated for Turkish, except for ESRS. All clinical assessments, except for the cognitive battery and the Effort Expenditure for the Rewards Task (EEfRT), were performed at two timepoints at least 6 months apart. The mean interval between the two interviews was 10.32 (2.56) months.

### 2.3. Effort Expenditure for the Rewards Task (EEfRT) 

EEfRT is a computer-based behavioral paradigm developed by Treadway et al. that assesses effort-based decision making by measuring how much physical effort individuals exert to obtain varying amounts of monetary rewards [15]. EEfRT was programmed in the Inquisit Millisecond software package 5 (https://www.millisecond.com/download/, accessed on 2 June 2019) and administered using Inquisit Player. In order to be consistent with the previous literature, we did not make any changes to the task. The original task consists of consecutive trials that require participants to choose between two difficulty levels (“hard task” and “easy task”). In each trial, participants are given the option to choose between easy and hard tasks. To complete the easy task, the participant had to press the specified key of the computer 30 times in succession with the index finger of the active hand within 7 s. A fixed 1 TRY was offered for each easy task. To complete the difficult task, the participant had to press the specified key of the computer 100 times in a row with the pinky finger of his passive hand within 21 s. Reward amounts ranging from 1.24 TRY to 4.30 TRY were offered for each difficult task. The amount of reward offered for the hard task differed in each trial, and, at the start of the trial, the participant was shown how much reward was provided for the hard task in that trial. There were three different probability levels for receiving the reward after successful completion of each trial: 88%, 50%, and 12%. These probability levels varied from trial to trial, and the level applicable to that trial applied to hard and easy tasks. There were equal proportions of tasks from all probability levels throughout the experiment. Probability levels were evenly distributed over the rewards for difficult tasks. All participants were offered the same randomized order of challenge reward amount. All trials began with a 5 s selection period, during which participants were shown the amount of reward they could earn for easy and difficult tasks, and the probability of winning the reward for that trial was shown. After the task was completed, a feedback screen appeared for 2 s, reporting whether the participant had completed the task or not. Then, if the participant had successfully completed the task, a second 2 s feedback screen appeared, stating whether the person was given the reward in that trial and, if so, how much reward money was given. At the beginning of the task, all participants were given instructions on how to play the task, and four test trials were completed. They were offered a fixed payment for their participation, plus additional payment depending on their performance on the task. Participants had 20 min to complete the entire task.

### 2.4. Statistical Analysis

The EEfRT was evaluated considering the percentage of total hard task selection across different probability (88%, 50%, and 12%) and reward magnitude levels (low, medium, and high). The reward magnitude was divided into three categories: low reward 1.24–2 TL, medium reward 2.01–3 TL, and high reward 3.01–4.12 TL. A 2 (group: SCZ and HC or PNS vs. non-PNS) × 3 (reward probability: 88%, 50%, and 12%) × 3 (reward magnitude: high, medium, and low reward) repeated-measures analysis of variance (ANOVA) test was used to investigate the main effects and interactions of probability level, reward magnitude, and diagnostic group on participants’ hard task choices. In the repeated-measures ANOVA test, the percentage of choosing the difficult task was the independent variable. Probability and reward levels, the dependent variables, were assigned as within-subject factors; and the diagnosis group was assigned as a between-subject factor. In cases where sphericity could not be achieved in factors with three levels, the Greenhouse–Geisser correction was applied. 

Pearson or Spearman correlation tests were used to analyze the association of clinical measurements with EEfRT performance, depending on the normality of the distribution as assessed by the Shapiro–Wilk test. The composite cognitive scores were calculated by averaging *z*-scores of individual cognitive tests. Then, *z*-scores were standardized on the basis of the cognitive scores of HC. Although the mean hard task selection rate in 88% probability trials, the mean hard task selection rate in high reward trials, the difference in hard task selection rate between 88% and 12% trials, and the difference in hard task selection rates of high and low reward trials were frequently used in the literature [15,19,20,21,23,24,29,52], due to the exploratory nature of this study, we used the mean hard task selection rate in all conditions including all levels of reward probability and reward magnitude. The statistical significance level was set at *p* < 0.05. Statistical analyses were performed using the IBM SPSS (Statistical Package for Social Sciences) program version 21.0 (IBM, Armonk, NY, USA).

A priori power analyses were conducted using G*Power Software version 3.1.9.6. (University of Kiel, Kiel, Germany) to determine the minimum sample size [53]. A total of forty participants were required for repeated-measures ANOVA with two groups and nine (3 × 3) measurements to achieve 80% power for detecting an effect size of 0.15 at 0.05 significance. As for correlations, 67 participants were required to achieve 80% power for detecting an effect size of 0.3 at 0.05 significance.

## 3. Results

### 3.1. Sociodemographic Variables

The groups did not differ in age, gender, or marital status, but there was a significant difference in education (*t* = 2.269; *p* = 0.027). The pairwise comparisons of sociodemographic and clinical variables between the study groups are presented in Table 1.

### 3.2. Results of SCZ vs. HC Comparison

In the EEfRT, the SCZ group chose the hard task in 31.13% of all trials (SD = 10.98), whereas HCs chose the hard task in 38.37% of all trials (SD = 10.34). None of the participants had a percentage of choosing the total difficult task above 90% or below 10%. No significant difference was observed in total trials attempted (SCZ: mean = 71.93, SD = 10.36; HC: mean = 75.53, SD = 7.80; *t* = 1.503; *p* = 0.134), but patients with SCZ completed significantly fewer trials compared to HCs (SCZ: mean = 63.93, SD = 9.49; HC: mean = 74.80, SD = 8.01; *t* = 3.593; *p* = 0.001). There was no significant difference in mean reaction time between the two groups (SCZ: mean = 2271.37, SD = 547.76; HC: mean = 2074.60, SD = 360.59; *t* = 1.635; *p* = 0.111).

#### 3.2.1. Main Effects

The repeated-measures ANOVA test indicated a statistically significant main effect of the group (F(1;50) = 10.801; *p* = 0.002; *p*η^2^ = 0.076), with SCZ engaged in overall less effortful choices compared to HC. Furthermore, the main effects of the reward probability (F(1.6;98) = 99.451; *p* = 0.0005; *p*η^2^ = 0.628) and the reward magnitude (F(1.4;86.2) = 166.47; *p* = 0.0005; *p*η^2^ = 0.738) were significant, which means that, overall, participants’ likelihood of choosing the hard task increased as the level of reward probability and reward magnitude increased.

#### 3.2.2. Group Effects and Interactions

**Figure 1:**
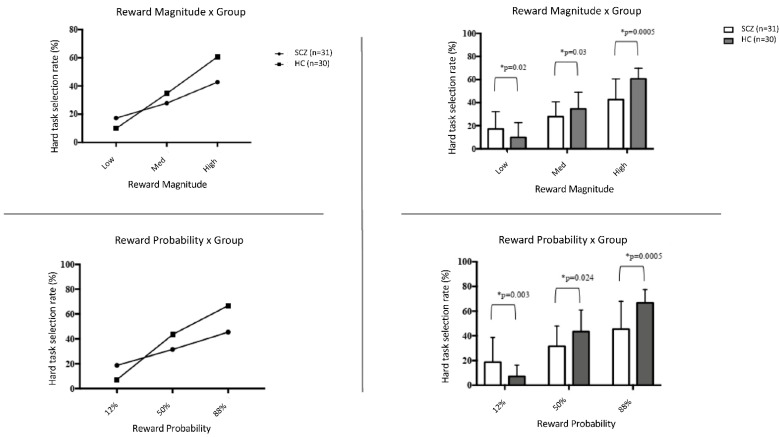
The proportions of hard task selection across patient groups in different reward probability and reward magnitude conditions. * Significance level at *p* < 0.05.

The group × reward probability interaction was statistically significant (F(1.66;98) = 16.192; *p* = 0.0001; *p*η^2^ = 0.215). Post hoc comparisons revealed that the SCZ group chose the hard task more in the 12% probability level compared to HC (F(1;59) = 9.337; *p* = 0.003; *p*η^2^ = 0.137), whereas HCs made more hard task choices compared to SCZ in the 88% and 50% probability levels (F(1;59) = 18.922; *p* = 0.0001; *p*η^2^ = 0.243 and F(1;59) = 5.388; *p* = 0.024; *p*η^2^ = 0.084, respectively). In both groups, the percentage of choosing the hard task increased as the reward probability increased (Figure 1). That is, the percentage of choosing the hard task was significantly different in the 12% to 50% (*p* = 0.002 in SCZ; *p* = 0.0005 in HC) and 50% to 88% comparisons (*p* = 0.0005 in SCZ; *p* = 0.0005 in HC) in both groups.

The group × reward magnitude interaction was also significant (F(1.46;86.21) = 19.861; *p* = 0.0005; *p*η^2^ = 0.252). Post hoc comparisons revealed that SCZ group made significantly more hard task choices in the low-reward-magnitude trials compared to HCs (F(1;59) = 5.715; *p* = 0.02; *p*η^2^ = 0.088), whereas HCs made more effortful choices in the medium- and high-reward-magnitude trials compared to the SCZ group (F(1;59) = 4.937; *p* = 0.03; *p*η^2^ = 0.077 and F(1;59) = 24.336; *p* = 0.0005; *p*η^2^ = 0.292, respectively). In both groups, the percentage of choosing the hard task increased as the reward magnitude increased. That is, the percentage of choosing the hard task was significantly different in comparisons of low to medium reward magnitude (*p* = 0.0001 in SCZ; *p* = 0.0001 in HC) and medium to high magnitude (*p* = 0.0001 in SCZ; *p* = 0.0001 in HC) in both groups. The group × reward probability × reward magnitude interaction was also statistically significant (F(2;100) = 1.693; *p* = 0.189; *p*η^2^ = 0.109).

### 3.3. The Association of Effort Allocation Capacity with NS

#### 3.3.1. Results of PNS vs. Non-PNS Comparison

Comparisons of the sociodemographic and clinical features of the patient groups can be found in Table 2. Patients with PNS attempted slightly more trials compared to patients without PNS (*t* = 2.389, *p* = 0.024). Nevertheless, there was no difference between the patient groups in the total number of trials completed (*t* = 1.547, *p* = 0.133). Overall, the patients with PNS chose the hard task in 25.06% (SD = 10.76) of the trials, whereas HCs chose the hard task in 35.52% (SD = 9.08) of the trials. The mean reaction time did not differ between the patient groups (PNS: mean = 2163.47 SD = 514.76; non-PNS: mean = 2347.53, SD = 572.72; *t* = 0.888; *p =* 0.374).

There were significant main effects of the group (F(1;25) = 11.108; *p* = 0.002; *p*η^2^ = 0.277), reward probability F(1.43;41.71) = 11.817; *p* = 0.0001; *p*η^2^ = 0.290), and reward magnitude (F(1.29;37.42) = 26.454; *p* = 0.0001; *p*η^2^ = 0.477) in the repeated-measures ANOVA analysis. However, there were no significant group × reward probability, group × reward magnitude, or three-way interactions.

#### 3.3.2. Correlations of EEfRT Performances with NS

Correlations with the NS total scores and motivation and pleasure (MAP) subdomain scores are presented in Table 3. The BNSS total score was significantly negatively correlated with the total rate of hard task selection, hard task selection rate at 50% reward probability, and hard task selection rate at medium reward magnitude. The SANS total score and the SNS total score were significantly correlated with the hard task selection rate at 50% reward probability, with the direction of correlation being negative and positive, respectively.

As for the correlations with the MAP subdomains, MAP subdomains of BNSS and SANS were significantly negatively correlated with the total rate of hard task selection and hard task selection rate at 50% reward probability. Additionally, the BNSS MAP subdomain was negatively correlated with the hard task selection rate at 88% and hard task selection rate in medium-reward conditions, whereas SANS MAP was negatively correlated with the hard task selection rate in the medium-reward condition. The MAP subdomain of SNS did not correlate with any EEfRT performance measures. No correlations were observed with the difference-score analyses (Supplementary Table S1).

### 3.4. Correlations with Other Clinical Parameters

No significant correlations were observed between any EEfRT measures and SAPS, CDSS, ESRS, and mean antipsychotic doses (Supplementary Table S2). Significant positive correlations were found between the composite cognition score and the total rate of hard task selection (*r* = 0.406, *p* = 0.032) and hard task selection rate at medium-reward levels (*r* = 0.382, *p* = 0.045). The PSP score was also positively correlated with the hard task selection rate at 50% (*r* = 0.394; *p* = 0.031).

## 4. Discussion

### 4.1. Main Findings

This study investigated effort-based decision-making differences between patients with recent-onset SCZ and HC. Furthermore, we examined the relationship between the effort allocation capacity and NS both continuously by using different NS scales and categorically by using the PNS distinction. Our findings suggested that patients with SCZ showed a general reduction in effort allocation for monetary rewards compared to HC, which was more pronounced in high- and moderate-probability and -magnitude trials. Secondly, we found that the NS, particularly amotivation, negatively correlated with effort expenditure when the magnitude of the reward and the possibility of getting the reward were moderate. Thirdly, patients with PNS showed a more significant reduction in effort allocation compared to patients without PNS.

### 4.2. SCZ vs. HC Comparison

When the participants’ choices under different conditions were examined in detail, we found that, while the reward magnitude and probability levels were medium and high, patients with SCZ chose the difficult task at a lower rate than HC. This difference between the two groups was especially evident when the reward magnitude and probability levels were highest. Unlike studies revealing reduced effort allocation only when the reward magnitude and probability were higher [23,24,31], we also observed a general reduction in the proportion of high-effort trials in patients with recent-onset SCZ compared to HC. Examples of such a group difference also exist in the literature [20,30]. In addition, we observed that patients with SCZ chose the hard task more often than HC in the low-probability and -magnitude trials, as also found in some previous studies. [19,20,21,24]. In other words, patients with SCZ preferred the easy task with low reward more in trials where it would be advantageous to exert more effort, but the hard task in trials where effort was expected to be strategically minimized. Overall, adding to the evidence in the literature, these findings suggest that patients with SCZ both have a general reduction in effort capacity and make inefficient choices in terms of effort allocation. It is important to note that, in our study, the percentage of choosing the hard task increased significantly with the increasing amount of reward and the probability of winning a reward in both groups. There are studies in the literature that found this trend only in HC [20]. However, the fact that the increase in the tendency to choose the hard task with the increase in the magnitude of rewards that can be won and the probability of winning the reward has also been observed in SCZ may indicate two possibilities. The first one is that the patients did not make arbitrary choices and were able to understand and apply the rules of the EEfRT task. The second one is that the reward valuation may at least partially be spared in SCZ as the patients were responsive to increasing levels of reward yet still were willing to exert less effort than HC. In line with this, a relatively preserved value-guided decision-making was found in previous studies [54].

### 4.3. Association of Effort Allocation Capacity with NS

One of the main aims of the present study was to investigate the relationship between effort allocation and NS using different types of NS assessments (old- vs. new-generation scales; clinician vs. self-report; cross-sectional vs. longitudinal assessment) and different EEfRT performances. Interestingly, apart from SNS-MAP, all scales and MAP subscales were correlated with hard task selection rate in medium-reward-magnitude and/or medium-reward-probability conditions. Only the MAP domain of BNSS was associated with the high-probability condition, whereas none of the NS scales or subscales correlated with the high-reward-magnitude, low-reward-magnitude, or low-reward-probability conditions. These findings may indicate that patients exhibit effort-related attitudes independent of NS in situations where it is more certain whether a reward will be obtained or not. Similarly, a rewarding stimulus of very high or very low potency may reduce the impact of NS on effort-based decision making. However, the more moderate precision and potency of the stimulus may cause people with NS to perform differently than those without NS. In the literature, very few studies considered moderate-level trials as a performance parameter [21,22]. Fervaha et al. (2013) found that apathy was significantly correlated to hard task selection rate in high-reward (50%) trials [21]. Additionally, similar to NS, there was a positive correlation between functioning and effort expenditure only in the 50% probability condition, which is in line with previous research that found an association between functioning and various EEfRT parameters [23,24,26]. It is known that NS, especially motivation/pleasure deficits, are closely related to functioning [6,7]. Overall, our results may indicate that, despite NS, sufficiently high-potency stimuli may trigger reward responses in people with schizophrenia. However, further studies investigating the effort-based attitudes in response to vague rewarding stimuli in patients with NS and functioning are needed.

As far as we know, this is the first study to evaluate effort-based decision making in the context of a longitudinal evaluation of NS in SCZ. Our results suggested that patients with PNS were less willing to exert effort than patients without PNS. Fervaha et al. (2015) also found a group-level difference in EEfRT performance between patients with and without deficit syndrome [29]. A critical difference was that the evaluation of persistence was cross-sectional and retrospective in the DS assessment, whereas it was prospective in the PNS assessment [55].

### 4.4. Comparisons of Different NS Measurements

In the comparison of NS scales, there was a clear difference between the scores of self-report and clinician-rated scales, as correlations between self-report scales and EEfRT parameters were very limited. This also supports a recent meta-analysis comparing self-reported, clinician-rated, and performance-based motivation measures in SCZ, although only two studies were included in the self-report vs. performance-based measure comparison [56]. On the other hand, in our study, none of the clinician-rated scales vastly outperformed the other. Overall, the correlations between the clinician-rated scale scores and EEfRT performance measures were low to medium. However, BNSS demonstrated a slightly more consistent association with effort allocation capacity, with more correlations (including one with high probability conditions) and more robust correlation coefficients for total scores compared to SANS. This difference was less pronounced in the correlations conducted with MAP subdomains. Conceptually, new-generation scales, which were developed after the NIMH-MATRICS Consensus Statement, provide a more detailed assessment of amotivation by separating internal experience from behavior and including aspects such as anticipatory and consummatory pleasure. In the literature, no correlation was found between SANS and EEfRT task measures [21,23,24], except for a trend-level association when covarying for medication dose [19]. Plus, there is an equal number of studies that did [27,28] and did not [25,57] find correlations with SANS in cognitive or physical effort exertion tasks other than EEfRT. We observed correlations between SANS and EEfRT scores only when the rewarding stimuli were vague. This may be due to the fact that other studies generally did not include correlations with medium-level conditions. The EEfRT studies that used BNSS were relatively few. In one study, BNSS and SANS were merged to obtain composite scores of avolition and anhedonia correlated with reward magnitude and probability differences [23]. Strauss et al. (2021) found a correlation between BNSS total and MAP subdomain scores and effort expenditure in the very-high-reward-magnitude condition in individuals at clinical high risk for psychosis [52]. In studies conducted with other cognitive or physical effort tasks and using BNSS, NS patients were found to be significantly associated with effort performance when considered continuous or categorical variables [27,31,58,59]. There was also a cognitive effort study in which no correlation was found when BNSS was used [57]. Another new-generation scale, CAINS, was also used in effort-based decision-making paradigms and resulted in significant correlations with task performances [20,26,60], although one study reported otherwise [22]. Putting all these together, the use of new-generation scales may enable a more accurate evaluation of NS in relation to effort-based decision making.

### 4.5. Strengths, Limitations, and Future Recommendations

The present study had some strengths. First, we recruited patients with recent-onset SCZ to reduce the confounding effects of the chronicity of the disease and prolonged medication exposure, which may have affected the effort allocation process. Secondly, we extensively investigated NS including different types of scales and a temporal approach by considering PNS. Furthermore, to minimize the secondary negative symptoms, we only included patients using second-generation antipsychotic medications. There were several limitations of the study that should be considered. Firstly, the sample size was small, especially after dividing the group with respect to their PNS statuses. Increasing the sample size would have increased the statistical power. Plus, we did not apply a correction for multiple comparisons because it was too restrictive considering the sample size. Future studies with more samples could use such corrections. Secondly, our participants were not medication-free. Although we only included patients on second-generation antipsychotics and did not find an association with medication dose, a possible contribution of antipsychotic medication cannot be excluded. Thirdly, the patient group was slightly less educated than HC, which is an expected phenomenon considering the diagnosis could impair the education process. Furthermore, in line with the original study, we did not individually calibrate the number of button presses during the EEfRT task. This might have led to lower task completion rates in individuals with motor impairments. However, Barch et al. (2014) demonstrated that the easy or hard task selection process was independent of finger tapping speed [23]. Regarding measurement tools, ESRS has not yet been validated in the Turkish language. Additionally, future studies could implement more direct measurement methods such as ecological momentary assessments (EMAs). Although the small number of existing studies regarding EEfRT and EMA gave contradictory findings [22,61], novel digital phenotyping methods can be promising in terms of effort expenditure research in patients with SCZ [62].

## 5. Conclusions

Our findings contribute to the existing literature suggesting that patients with SCZ may show a general reduction in effort allocation capacity and make inefficient choices in terms of effort allocation, although they are not totally reward-insensitive. The effects of NS on effort expenditure can be more pronounced in situations where the probability or the magnitude of the effort is moderate. Future studies are needed to evaluate the relationship among the real-life correspondences of NS, effort expenditure for the rewards, and reward valuation.

## Figures and Tables

**Table 1:** Sociodemographic, cognitive, and clinical characteristics.

	SCZ (*n* = 31)	HC (*n* = 30)	Test Statistics (*t*, χ^2^)	*p*-Value	PNS (+) (*n* = 13)	PNS (−) (*n* = 18)	Test Statistics (*t*, χ^2^)	*p*-Value
Sociodemographic characteristics								
Age, years	25.45 (5.46)	26.00 (2.44)	0.503	0.614	23.30 (4.8)	26.63 (5.55)	1.746	0.091
Gender, % female	8 (25.8)	8 (26.7)	0.006	0.939	2 (15.4)	6 (33.3)	0.412	0.242
Education, years	12.32 (3.00)	13.83 (2.10)	2.269	0.027 *	11.00 (2.70)	13.21 (2.83)	2.203	0.035 *
Cognitive assessment								
TMT-B	119.21 (63.96)	60.25 (21.24)	4.061	<0.001 *	153.45 (72.73)	97.66 (46.28)	2.278	0.038 *
CFT	15.60 (3.17)	22.48 (3.87)	7.275	<0.001 *	14.09 (6.94)	16.57 (3.22)	2.223	0.035 *
DSST	55.85 (19.61)	87.50 (14.09)	6.933	<0.001 *	47.72 (61.50)	19.10 (18.12)	1.946	0.062
Clinical characteristics								
Age at onset, years	22.71 (5.5)	-	-	-	20.46 (4.33)	23.94 (5.87)	1.824	0.078
Duration of illness, years	2.93 (1.19)	-	-	-	3.07 (1.32)	2.78 (1.08)	0.674	0.505
OLZ equivalent doses, mg	17.07 (7.88)	-	-	-	21.86 (7.32)	13.9 (6.55)	3.150	0.004 *

* *p* < 0.05. CFT, Category Fluency Test; DSST, Digit Symbol Substitution Test; HC, healthy controls; OLZ, olanzapine; PNS, persistent negative symptoms; SCZ, schizophrenia; TMT-B, Trail Making Test-B.

**Table 2:** Mean clinical scale scores and their comparisons between patients with and without PNS.

	SCZ (*n* = 31)	PNS(+) (*n* = 13)	PNS(−) (*n* = 18)	Test Statistics (*t*)	*p*-Value
BNSS					
BNSS Total	38.82 (15.90)	44.53 (8.21)	29.00 (11.91)	4.298	<0.0001 *
BNSS MAP	28.04 (10.10)	29.38 (5.14)	19.11 (8.35)	4.145	<0.0001 *
BNSS ED	10.82 (7.52)	12.00 (5.35)	8.11 (4.59)	2.091	0.047
SANS					
SANS Total	47.81 (18.93)	52.41 (12.10)	38.50 (14.43)	2.752	0.010 *
SANS MAP	27.32 (8.76)	33.00 (4.61)	23.22 (8.82)	4.001	<0.0001 *
SANS ED	16.77 (8.74)	19.75 (9.19)	14.77 (8.07)	1.563	0.129
SNS					
SNS Total	24.78 (8.26)	17.84 (6.68)	27.68 (7.35)	3.853	0.001 *
SNS MAP	14.84 (5.29)	11.61 (4.25)	17.05 (4.84)	3.271	0.003 *
SNS ED	9.88 (3.27)	8.75 (3.46)	10.9 (2.81)	1.727	0.098
SAPS	12.0	12.0 (12.44)	9.16 (8.06)	0.719	0.481
CDSS	1.33 (1.09)	2.53 (3.01)	1.72 (1.56)	0.983	0.334
ESRS	6.33 (4.67)	7.38 (3.30)	5.77 (3.07)	1.391	0.175
PSP	46.25 (16.03)	40.76 (15.52)	51.38 (14.83)	1.929	0.064

* *p* < 0.05. BNSS, Brief Negative Symptoms Scale; CDSS, Calgary Depression Scale for Schizophrenia; ED, expressive deficits; EEfRT, Effort Expenditure for the Rewards Task; ESRS; Extrapyramidal Symptoms Rating Scale; MAP, motivation and pleasure deficits; NS, negative symptoms; PNS, persistent negative symptoms; PSP, Personal and Social Performance Scale; SANS, Scale for the Assessment of Negative Symptoms; SAPS, Scale for the Assessment of Positive Symptoms; SCZ, schizophrenia; SNS, Self-Evaluation of Negative Symptoms Scale.

**Table 3:** Correlations of EEfRT performance measures with different negative symptoms scale scores.

Variables	88%	50%	12%	High Reward	Mid Reward	Low Reward
NS Total Scores						
BNSS	−0.313	−0.497 **	−0.191	−0.309	−0.528 **	−0.184
SANS	−0.325	−0.368 *	−0.032	−0.253	−0.352	−0.055
SNS	0.051	0.360 *	0.132	0.102	0.284	0.086
MAP Subdomain						
BNSS	−0.434 *	−0.477 **	−0.152	−0.250	−0.462 *	−0.163
SANS	−0.258	−0.496 **	−0.030	−0.357	−0.453 *	−0.068
SNS	−0.102	0.245	0.138	0.007	0.093	−0.032

* *p* < 0.05, ** *p* < 0.01. BNSS, Brief Negative Symptoms Scale; EEfRT, Effort Expenditure for the Rewards Task; MAP, motivation and pleasure deficits; NS, negative symptoms; SANS, Scale for the Assessment of Negative Symptoms; SNS, Self-Evaluation of Negative Symptoms Scale.

## Data Availability

The data that support the findings of this study are available from the corresponding author (E.I.G.) upon reasonable request.

## Supplementary Materials

The following supporting information can be downloaded at: https://www.mdpi.com/article/10.3390/jcm11175060/s1, Table S1. Correlations of EEfRT difference measures with different negative symptoms scale scores. Table S2. Correlations of EEfRT performance measures with other clinical measures.
