# Supplementary material for: Impaired Effort Allocation in Patients with Recent-Onset Schizophrenia and Its Relevance to Negative Symptoms Assessments and Persistent Negative Symptoms
Source: J Clin Med. 2022 Aug 28;11(17):5060. doi: 10.3390/jcm11175060 (PMC9457458; doi:10.3390/jcm11175060)
Supplement: Supplementary file 1 [file jcm-11-05060-s001.zip › jcm-1875185-SI.pdf]

Supplementary Files:

Table S1. Correlations of EEfRT difference measures with different negative symptoms scale scores

Variables	88-12% Probability Difference	High-Low Reward Difference
NS Total Scores		
BNSS Total	-0.059	-0.192
SANS Total	-0.145	-0.254
SNS-Total	0.025	0.032
MAP subdomain		
BNSS	0.011	-0.191
SANS	-0.185	-0.332
SNS	0.057	0.072

BNSS, Brief Negative Symptoms Scale; EEfRT, Effort Expenditure for the Rewards Task; MAP, motivation and pleasure deficits; NS, negative symptoms; SANS, Scale for the Assessment of Negative Symptoms; SNS, Self-Evaluation of Negative Symptoms Scale.

Table S2. Correlations of EEfRT performance measures with other clinical measures

Variables	88%	50%	12%	High Reward	Mid Reward	Low Reward
SAPS Total	-0.024	-0.060	0.023	0.138	-0.091	-0.336
CDSS	0.253	-0173	-0.249	-0.007	0.007	-0.030
ESRS	-0.333	-0.090	-0.115	-0.029	-0.289	-0.149

Cognition (composite score)	0.253	0.194	0.055	0.238	0.382*	0.104
PSP	0.175	0.394*	0.129	0.330	0.289	-0.209
OLZ equivalent doses,	-0.202	0.052	0.175	0.034	-0.229	-0.223

* Correlations were significant at the 0.05 level. CDSS, Calgary Depression Scale for Schizophrenia; EEfRT, Effort Expenditure for the Rewards Task; ESRS; Extrapyramidal Symptoms Rating Scale; OLZ, olanzapine; PSP, Personal and Social Performance Scale; SAPS, Scale for the Assessment of Positive Symptoms.
